# Substantial Impact of School Closure on the Transmission Dynamics during the Pandemic Flu H1N1-2009 in Oita, Japan

**DOI:** 10.1371/journal.pone.0144839

**Published:** 2015-12-15

**Authors:** Shoko Kawano, Masayuki Kakehashi

**Affiliations:** 1 Graduate School of Health Sciences, Hiroshima University, Hiroshima, Japan; 2 Institute of Biomedical & Health Sciences, Hiroshima University, Hiroshima, Japan; The University of Tokyo, JAPAN

## Abstract

**Background:**

School closure is considered as an effective measure to prevent pandemic influenza. Although Japan has implemented many class, grade, and whole school closures during the early stage of the pandemic 2009, the effectiveness of such a school closure has not been analysed appropriately. In addition, analysis based on evidence or data from a large population has yet to be performed. We evaluated the preventive effect of school closure against the pandemic (H1N1) 2009 and examined efficient strategies of reactive school closure.

**Materials and Methods:**

Data included daily reports of reactive school closures and the number of infected students in the pandemic in Oita City, Japan. We used a regression model that incorporated a time delay to analyse the daily data of school closure based on a time continuous susceptible-exposed-infected-removed model of infectious disease spread. The delay was due to the time-lag from transmission to case reporting. We simulated the number of students infected daily with and without school closure and evaluated the effectiveness.

**Results:**

The model with a 3-day delay from transmission to reporting yielded the best fit using *R* ^2^ (the coefficient of determination). This result suggests that the recommended period of school closure is more than 4 days. Moreover, the effect of school closure in the simulation of school closure showed the following: the number of infected students decreased by about 24% at its peak, and the number of cumulative infected students decreased by about 8.0%.

**Conclusions:**

School closure was an effective intervention for mitigating the spread of influenza and should be implemented for more than 4 days. School closure has a remarkable impact on decreasing the number of infected students at the peak, but it does not substantially decrease the total number of infected students.

## Introduction

School closure is one of the important non-pharmaceutical interventions (NPIs) used to mitigate the spread of influenza. An ideal intervention should result in a delay in the peak incidence or a reduction in the number of infected students at the peak and decrease the total number of cases [1, 2]. For pandemic influenza, NPIs such as school closure are important strategies [3]. NPIs can potentially provide time to prepare for the production and distribution of a vaccine and antiviral medication as the preventive methods. The appropriate implementation of NPIs may decrease the maximum capacity of health care services required [4, 5]. In addition, because the spread of influenza in a community is strongly influenced by the outbreak among schoolchildren, school closure is an important strategy [6].

There are two types of school closures. First, a proactive closure is implemented to slow down the spread of influenza for the wider community on early onset. Second, a reactive school closure is implemented when many children and staff are infected. Simulation and empirical studies of pandemic influenza have reported that school closure mainly reduced the peak incidence, while only slightly reducing the cumulative infection attack rate [7–9]. Detailed strategies such as timing and duration should be considered depending on the severity of the influenza strain and the degree of the basic reproductive number [10–12]. In Japan, many reactive school closures (i.e., class, grade, and school closures) are implemented against seasonal influenza outbreak annually under the School Health Law. However, because the optimal timing and duration should be based on the specific influenza and local situation, the law does not specify the timing and duration of such closures. Therefore, each principal is responsible for the timing, duration, and level (i.e., class or grade) of the closure, while considering the estimated effect on lessons and school events. Principals usually decide the school closure strategies following discussions with the board of education, school nurses, and doctors [13].

In Japan, different guidelines were used for the school closures in the early and late phases against the pandemic (H1N1) 2009. Initially, in the early phase when the first case was confirmed, the basic strategy was proactive school closure [14]. The first proactive school closure occurred simultaneously in Hyogo and Osaka prefectures in May 2009. The school closure was considered effective for preventing the spread of influenza [15, 16]. Although the spread of influenza was temporally mitigated after the school closure, it resurged, with confirmation of many cases throughout Japan from mid-June [17, 18]. Consequently, many prefectures imposed detailed, but downgraded, rules for school closure against the pandemic that specified the timing and duration of the closure; while these rules were consistent within a prefecture, they differed between prefectures [19]. Subsequently, the Ministry of Health, Labour, and Welfare issued a new guideline for reactive school closure [20], although the recommendations remained almost the same as those in the previous guideline. Accordingly, the number of school closures in the 2009/2010 season approximately doubled compared with that in the 2008/2009 season [21].

Several studies have reported the effect of reactive school closure during the pandemic (H1N1) 2009 [22–25], concluding that at least 5 days are required [22, 23]. Although reactive closures are considered effective for reducing absenteeism within a class, the influence of school closure on the number of infected students in the entire population was still unclear [22–25].

The purpose of this study was to estimate the impact of school closure, considering the effect of the time delay as the latent period, by analysing the data of the pandemic (H1N1) 2009 in Oita City, Japan. The data were ideal to accomplish this purpose. First, the data were collected daily so that we could analyse the effect of the latent period in shorter intervals. Second, the proportion of schools surveyed among the total in our data was very high, resulting in data for more than 98% of elementary and junior high school students. Third, because it was a novel influenza and no vaccine was available, vaccine coverage did not influence the data. Therefore, all of the students could be considered susceptible. During the season, 96% of the influenza viruses isolated in Japan were the AH1pdm virus [26]. We also considered the influence of absolute humidity (AH), which is known to be causally associated with the onset of an epidemic [27–33].

## Materials and Methods

### Data collection

Our data included the number of infected students and school closures from August 2009 to March 2010 in Oita City of western Japan. We obtained the data directly from the Oita Prefectural Board of Education and Oita City Board of Education. Every public school was obligated to report the numbers of infected students, including the suspected cases, and the implementation of school closure by class. Every newly infected student and closure was reported daily to the respective board of education. Almost all of the reported students were diagnosed as infected with influenza by physicians using rapid diagnosis tests, and the false negative cases were closely monitored. In our study, the number of students at any level of closure, i.e., class, grade, or whole school, was calculated to precisely estimate the impact of school closure.

Oita City is located west of Kyushu Island and has a population of approximately 450,000 people. It is 400 kilometres from Hyogo and Osaka, where the first case in Japan was confirmed. As Oita City is surrounded by mountains and the sea and separated from other regions geographically, the effect of entry and exit populations was considered minimal. Approximately 96% of students were attending school and living within Oita City [34]. There were 134 public schools and 51,872 students (kindergarteners: 2.6%, elementary school students: 52.8%, junior high school students: 25.7%, and high school students: 18.7%). The trend of infection in the entire city was analysed using reports from the public schools because there were only 40 private schools (3 junior high schools, 27 kindergartens, and 9 high schools, with no elementary school). The age distribution of Japanese school children is 4–6 years for kindergarten, 7–12 years for elementary school, 13–15 years for junior high school, and 16–18 years for high school. Specifically, school closures included class, grade, and whole school closures. During this year, the rule for the timing of closures in Oita City was as follows: the class would be closed when infected students reached about 10% of the class, grade would be closed when multiple class closures occurred in the same grade, and whole school would be closed when multiple grade closures occurred in the same school. According to this rule, principals had to maintain closure for 4 days, starting the day after the infected threshold was exceeded. If newly infected individuals were identified during and after the school closure, the closure was extended by 1 more day. A class was still opened when only one or two cases were confirmed in the class. However, children were suspended from school until 2 days after their fever was alleviated under the School Health Law. This rule was established on August 20, 2009 by the Oita Prefectural Board of Education and adopted from the start of the second semester (September through December).

In addition to simple mechanistic transmission dynamics, we considered AH as one parameter of the epidemic. AH is related to the survival rate of the influenza virus and the significant effect of the epidemic [27–33, 35]. We collected meteorological data for Oita City from the Japan Meteorological Agency website [36]. We calculated AH (g/m^3^) from the average temperature (°C) and average relative humidity (%) for each day. AH was calculated by $AH=217\times E\times \frac{RH}{100}\times \frac{1}{T}$ and *E* = 6.11 × 10^7.5(*r*−273.3)^, where *E* indicates the mean saturation vapour pressure, *RH* indicates the relative humidity and *T* indicates an absolute temperature (*K*).

We obtained sentinel data to compare the number of cases of infected schoolchildren with other ages of people in Oita City. We were able to determine the weekly number of cases per clinic by age group. In Oita City, 16 outpatient clinics were registered in the influenza sentinel surveillance system. Data were provided by the Oita City Health Care Centre from September 2009 to March 2010. We illustrated the trend of cases of influenza-like illness by age group.

The data that were provided directly from the Oita Prefectural Board of Education and Oita City Board of Education were collected as secondary data sources, and we followed the ethical guidelines for epidemiological research performed in Japan. The data were anonymized and de-identified prior to analysis.

### Transformation for data analysis

The dataset of the number of infected students used in the analyses was the one transformed by the moving averages method. The raw data showed a weekly pattern. The reported numbers were large on Mondays and very small on school holidays (Saturdays and Sundays). To address weekend bias in the reported data, we calculated a moving average for 7 days; as a result, we obtained smoother data and eliminated the weekly periodicity. However, on Tuesday November 24, 2009, an extraordinarily large number of infected students were reported because of a successive three-day holiday. We could not obtain smooth data for the week by the moving average method only. Therefore, we adjusted the values of the week using the proportions of the previous week. Additionally, a time lag in reporting did not occur because our data were collected only on the days when students were absent.

We analysed data from September 1, 2009 and treated infected students as recovered students before that time because August was summer vacation. The rule of school closure was adopted from September 1, 2009.

### Basic structure of the model

We used a regression model based on the time continuous susceptible-exposed-infected-removed (SEIR) model. For simplicity, we ignored death and a change in school by students, because the interval of analysis was so short that such effects might not significantly influence the results. In addition, although children contact their friends during school closure [37, 38], the model did not involve such possible transmission during the school closure. This might have prevented an overestimated effect of school closure. Furthermore, some infected students are infectious without showing any symptoms. Our model was based on the hypothesis that the infected students were all symptomatic. Therefore, the actual number of infected students is unknown. In our model, the infectiousness of the symptomatic students was overestimated to account for infection transmitted by asymptomatic students. We calculated the total numbers of infected ($\hat{I}$), recovered ($\hat{R}$), and susceptible ($\hat{S}$) cases in the whole city according to a daily report from each school. The variable $\hat{I}(t)$ was the sum of the reported infected students on a particular day *t*, $\hat{R}(t)=\sum_{\tau =1}^{t-1}\hat{I}(\tau )$ and $\hat{S}(t)=N-\hat{I}(t)-\hat{R}(t)$. The number of exposed (*E*) was not explicitly counted and therefore was included in the number of $\hat{S}$.

**Fig 1:**
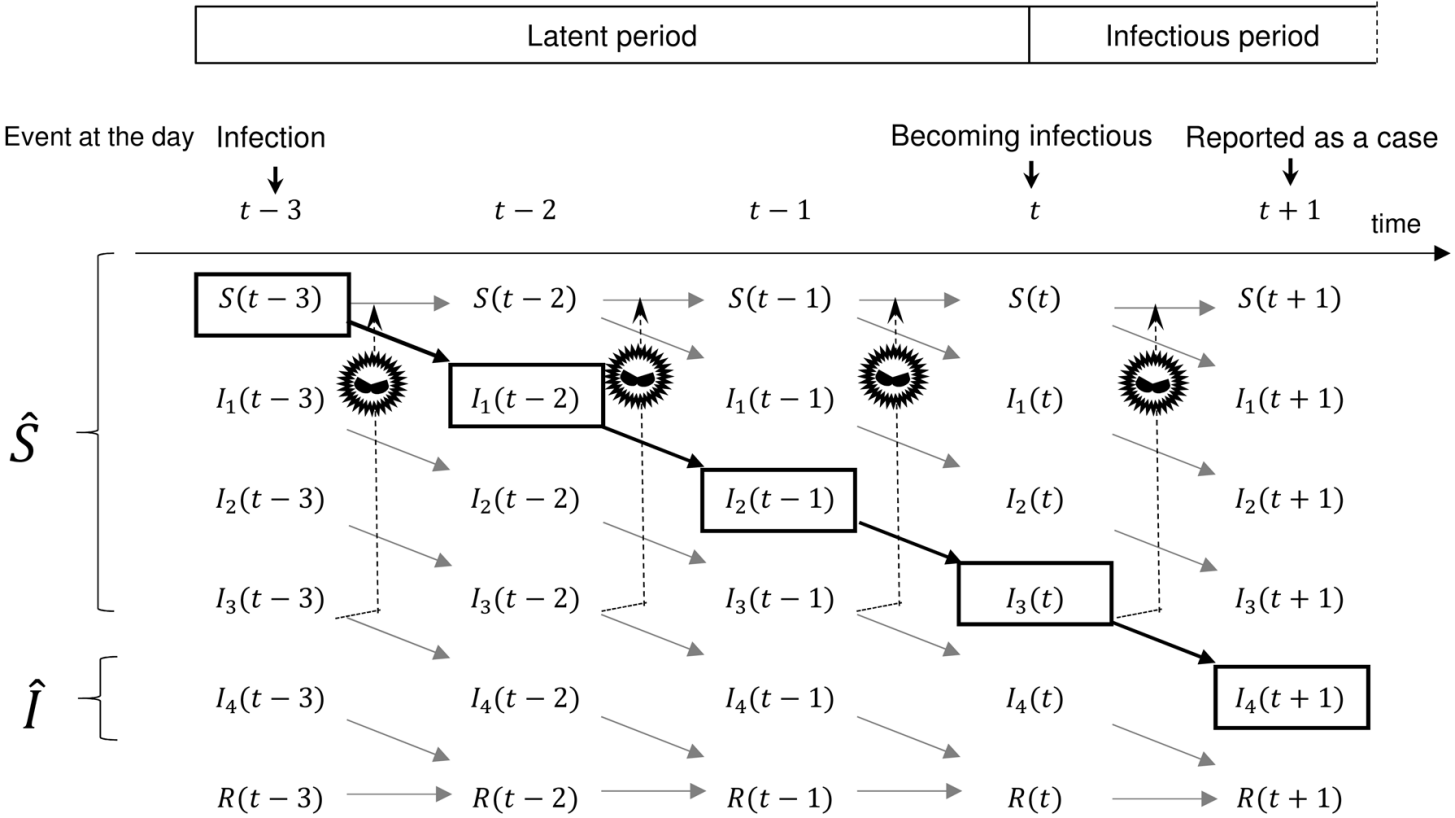
Structure of the transmission model that incorporates a time delay. Diagram of the relationship between the day of infection, the day of becoming infectious, and the day a case is reported, in the case of *d* = 3. The symbol of a virus indicates transmission. $\hat{S}$ and $\hat{I}$ are observed values of daily reports. *S* and *I* are theoretical values. *I*_4_(*t* + 1) is derived from *S*(*t* − 3), infected by *I*_3_(*t* − 3), becomes infectious at *t*, and is reported as a case at (*t* + 1). *I*_1_ and *I*_2_ indicate exposed students (i.e., *I*_1_ = *E*_1_ and *I*_2_ = *E*_2_). *I*_4_ is not infectious because it is absent from school.

We denoted the delay due to the latency period of infection by *d*. We considered that we only knew the reported numbers of cases and that infectious disease epidemics have an incubation and latent period. The beginning of the infectious period starts 1 day earlier than the onset of symptoms [39]. We carefully considered the duration between infection, the infectious period, and the day of the report. In Fig 1, we show the transmission model in the case of *d* = 3. The reported numbers of susceptible students ($\hat{S}(t)$) and infected students ($\hat{I}(t)$) are not the same as the theoretical values (*S*(*t*) and *I*(*t*)). In Fig 1, *I*_4_(*t*) is the number of students reported as infected, $\hat{I}(t)$. The transition from *I*_*i*_(*t*) to *I*_*i*+1_(*t*+1) instantly occurs in one time step (i.e., 1 day) for *i* < 4. The variables *I*_1_(*t*), *I*_2_(*t*), and *I*_3_(*t*) represent pre-symptomatic students. Although *I*_3_ is infectious, neither *I*_1_ nor *I*_2_ is infectious and can be written as *E*_1_ and *E*_2_. *I*_4_(*t*) is not infectious because it is absent from school. Infected students are counted as susceptible students before they are reported, i.e., *S*(*t*) represents the theoretical number of susceptible students. Thus, $\hat{S}(t)=S(t)+I_{1}(t)+I_{2}(t)+I_{3}(t)=S(t-3).$ Therefore, $\hat{I}(t+1)$, i.e., *I*_3_(*t*) infects *S*(*t*) at *t*. In the SEIR model, $\hat{S}$, $\hat{I}$, and $\hat{R}$ are given by the following:

$$\hat{S}(t+1)=\hat{S}(t)\exp\left(-\beta (t-d)\hat{I}(t-d+1)\right),$$

$$\hat{I}(t+1)=\hat{S}(t)\left\{1-\exp\left(-\beta (t-d)\hat{I}(t-d+1)\right)\right\},$$

and

$$\hat{R}(t+1)=\hat{R}(t)+\hat{I}(t).$$

Then,

$$\hat{I}(t+1)=\hat{S}(t)\left\{1-\exp\left(-\beta (t-d)\hat{I}(t-d+1)\right)\right\}≅\beta (t-d)\hat{S}(t)\hat{I}(t-d+1).$$

In data analyses, we used a generalized discrete SEIR model to estimate the number of infected students and added AH as a parameter related to the spread of infection. In our generalized model, newly infected students are given by the following.

$$\log_{10}\hat{I}(t+1)=\beta_{0}+\log_{10}\hat{S}(t)+\log_{10}\hat{I}(t-d+1)+\beta_{1}AH(t-d)$$ (1)

Using common logarithms of the values, if the observed value was 0, we replaced it with 0.5.

We were able to determine *β* values by regression analysis. In regression analysis, we used values observed before winter vacation. Different values of the parameters should be suitable for winter vacation.

Students isolated by school closure should not be exposed to the risk of infection. Therefore, susceptible students were divided into subjects in opened schools, *SO*_*d*_(*t*), and subjects in closed schools, *SC*_*d*_(*t*), at day *t*. *SO*_*d*_(*t*) and *SC*_*d*_(*t*) are given by: $SO_{d}(t)=\sum_{i,j}\left(1-C_{ij}(t-d)\right){\hat{S}}_{ij}(t)$ and $SC_{d}(t)=\sum_{i,j}C_{ij}(t-d){\hat{S}}_{ij}(t)$. Here, *C*_*ij*_ is the information on school closure: if the school is open, *C* = 0 and if the school is closed, *C* = 1; *i* is the school number, *i* = 1,2…, *i*_max_, and *j* is the class number, *j* = 1,2…, *j*_max_ (*i*). *SO*_*d*_(*t*) and *SC*_*d*_(*t*) are dependent on *d* because they are calculated from $\hat{S}(t)$ using *C*(*t* − *d*). Therefore, newly infected students during school closure are given in Eq (1) by replacing $\hat{S}(t)$ by *SO*_*d*_(*t*):

$$\log_{10}\hat{I}(t+1)=\beta_{0}+\log_{10}SO_{d}(t)+\log_{10}\hat{I}(t-d+1)+\beta_{1}AH(t-d)$$ (2)

#### Simulation under the implementation of school closure

Simulation for the number of infected students under school closure is given by Eq (2). We estimated *SO*_*d*_(*t*) for the simulation. We used the statistical relationship between *SC*_*d*_(*t*) and $\hat{I}(t)$ to calculate *SO*_*d*_(*t*) as follows.

$$\log_{10}SC_{d}(t)=\beta_{2}+\beta_{3}\log_{10}\hat{I}(t)$$ (3)

and

$$SO_{d}(t)=\hat{S}(t)-SC_{d}(t)$$ (4)

We were able to determine the *β*_0_–*β*_3_ values by regression analysis from the observed values. For another simulation without school closure, the numbers of infected students are given by Eq (1) by replacing *SO*_*d*_(*t*) with $\hat{S}(t)$.

We used *R* ^2^ (the coefficient of determination) to evaluate the goodness-of-fit of the regression model, i.e., Eq (2). Pearson’s correlation coefficient between observed and simulated values of students infected daily or the cumulative number of infected students was used to evaluate the most appropriate delay, *d*.

## Results

### Summary of pandemic (H1N1) 2009

**Fig 2:**
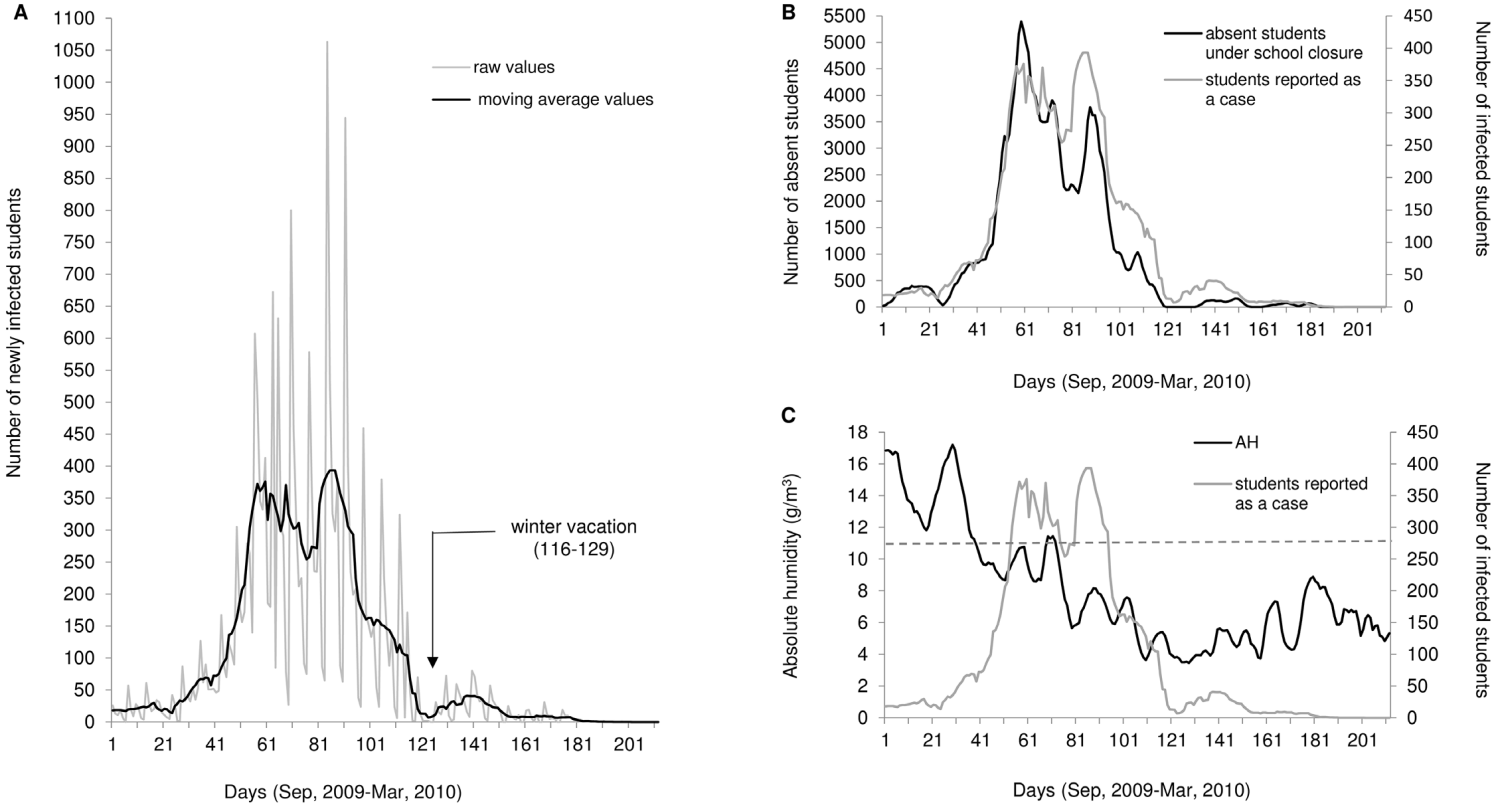
Time course of pandemic (H1N1) 2009 in Oita City, Japan. From September 2009 to March 2010, (A) a comparison of infected raw values of students and the moving average for 7-day values, (B) comparison of the number of absent students under school closure and students reported as a case, and (C) comparison of absolute humidity (AH) and the number of students reported as a case. The grey dashed line indicates AH of 11 g/m^3^. The number of absent students under school closure, number of students reported as a case, and AH are shown as a moving average for 7 days.

The graph of the numbers of infected students shows two notable peaks using a moving average (Fig 2A). The number of infected students began to increase gradually from the end of September 2009. The first peak was at the end of October 2009, and the second peak was at the end of November 2009. The number of infected students on the first peak was almost the same as that of the second peak. The curve of absent students also had two notable peaks (Fig 2B). The two peaks were located at nearly the same time as the peaks of infected students. A highly significant positive correlation was observed between the numbers of reported cases and absent students under school closure (*r* = 0.964, p < 0.001).

During the first peak, the number of absent students corresponded to the number of infected students, and during the second peak, the number of absent students were not as large as the number of infected students. AH and the number of infected students by time are shown in Fig 2C. Shoji reported AH below 11 g/m^3^ at the onset of influenza epidemics in Japan [40]. Horizontal dashed line in Fig 2C indicates the AH of 11 g/m^3^. AH decreased below 11 g/m^3^ at the end of September 2009. This timing is the same as the beginning of the increase in the number of infected students. AH was almost maintained at < 11 g/m^3^ after the beginning of October 2009.

**Fig 3:**
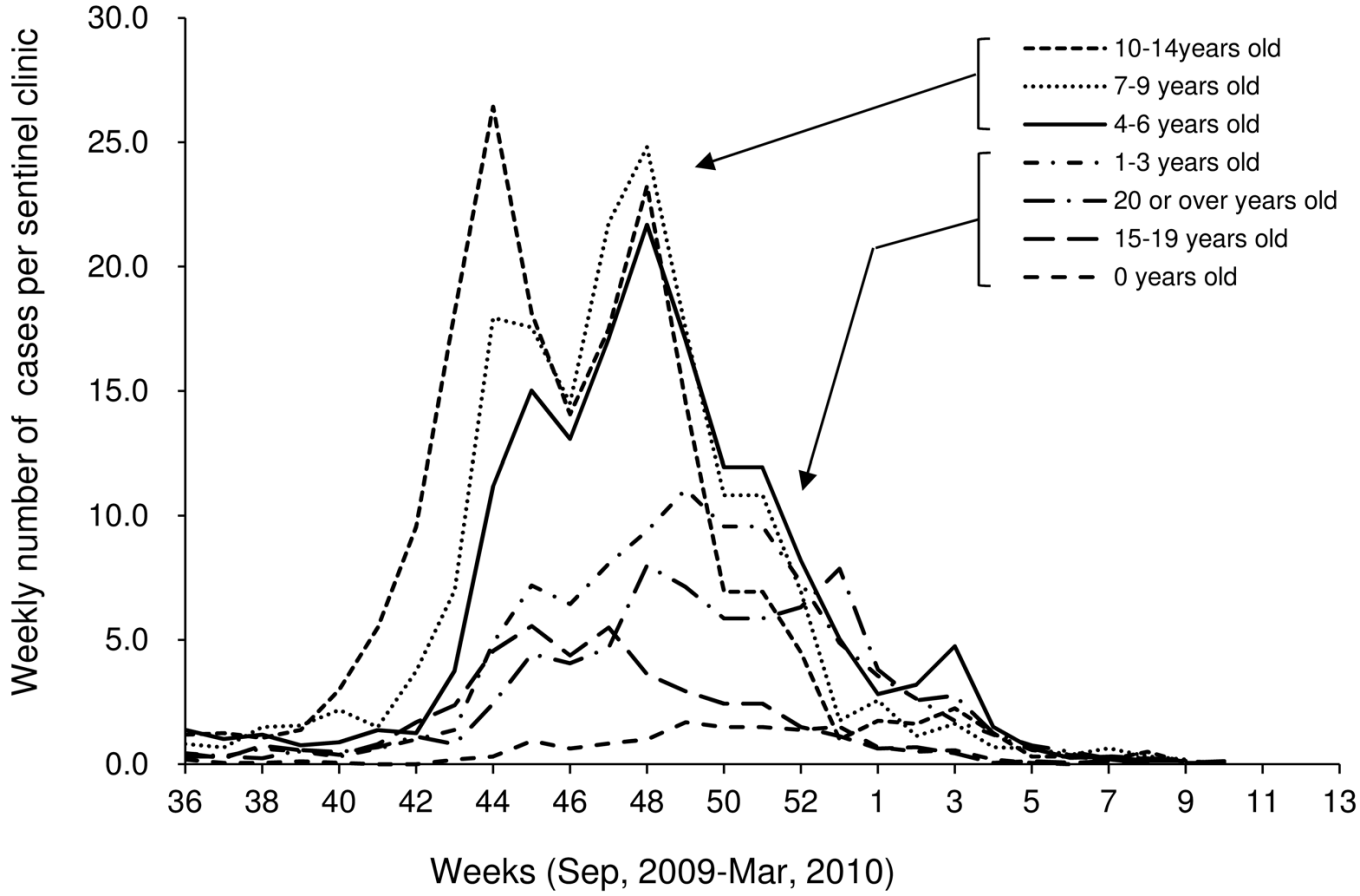
Sentinel reports by age group in Oita City, Japan from September 2009 to March 2010. Data were collected from 16 randomly chosen hospitals in Oita City and provided by Oita City Health Care Centre. The general age distribution in Japanese schools is 4–6 years for kindergarten, 7–12 years for elementary school, 13–15 years for junior high school, and 16–18 years for high school students.

In Fig 3, we show an overview of the weekly number of cases from sentinel reports of influenza by age group in Oita City in pandemic (H1N1) 2009. Obviously, the number of cases among children aged 4–6 years, 7–9 years, and 10–14 years is larger than the number of cases in individuals of other ages; moreover, cases in these age groups occurred earlier than those in other age groups. Moreover, the number of children aged 7–9 years and 10–14 years had two remarkable peaks. The highest number of cases occurred in week 44 for those aged 10–14 years, which was the first peak. Children aged 10–14 years were senior students and elementary and junior high school students. The highest number of cases occurred in week 48 for those aged 7–9 years, which was the second peak. Children aged 7–9 years were early elementary school students. The highest number of cases occurred in week 49 for adults. The curves of other age groups, except for schoolchildren, had less clear peaks than that of the school aged children group.

### Estimation of the partial regression coefficients

The results of regression analysis with different time delay based on the Eq (2) that predicts the number of infected students are shown in the left hand columns in Table 1. The results of regression analysis based on the Eq (3) that predicts the number of susceptible students under school closure are shown in the right hand columns. The goodness-of-fit of all cases was close to 1 (*P* < 0.001). In partial regression coefficients of predicting infected students, as the delay was longer, the value of *β*_1_ became closer to 1: i.e., the influence of absolute humidity became smaller.

**Table 1:** Goodness-of-fit of the model and partial regression coefficients in different time delay.

Delay	Predicting infected students			Susceptible students under school closure		
	Partial regression coefficients			Partial regression coefficients		
	*β*_0_	*β*_1_	*R* ^2^ ^†^(*P* Value)	*β*_2_	*β*_3_	*R* ^2^ (*P* Value)
*d* = 1	-4.461	-0.016	0.994 (*P* < 0.001)	0.855	0.979	0.738 (*P* < 0.001)
*d* = 2	-4.477	-0.013	0.989 (*P* < 0.001)	0.763	1.015	0.774 (*P* < 0.001)
*d* = 3	-4.490	-0.011	0.985 (*P* < 0.001)	0.677	1.049	0.764 (*P* < 0.001)
*d* = 4	-4.502	-0.009	0.979 (*P* < 0.001)	0.497	1.124	0.698 (*P* < 0.001)
*d* = 5	-4.518	-0.007	0.971 (*P* < 0.001)	0.319	1.198	0.659 (*P* < 0.001)

The meaning of partial regression coefficients in predicting infected students are shown in Eq (2). *β*_0_ is the intercept, and *β*_1_ represents the coefficient of absolute humidity (AH). The meaning of partial regression coefficients in susceptible students under school closure is shown in Eq (3). *β*_2_ is the intercept, and *β*_3_ represents the coefficient of the number of infected students.

^†^ *R* ^2^ includes the results of not only Eq (2) but also Eqs (3) and (4).

### Simulation under the implementation of school closure

**Fig 4:**
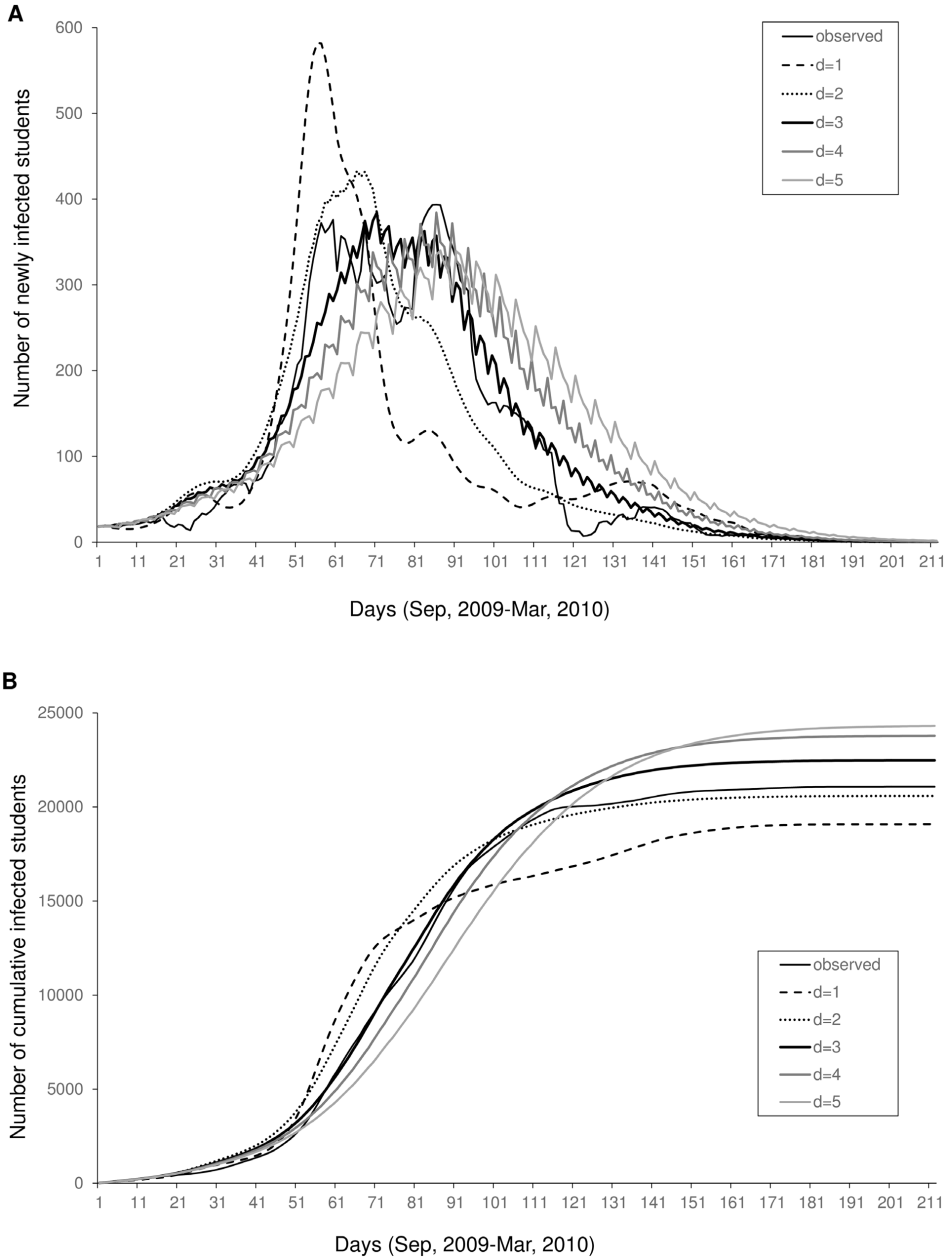
Simulated time course with various degrees of delay *d* = 1–5 in Oita City, Japan. From September 2009 to March 2010, (A) the number of newly infected students and (B) cumulative number of infected students. The parameters we used for each *d* are shown in Table 1.

Simulation curves of the number of infected students and the cumulative number of infected students for *d* = 1–5 are shown in Fig 4. The epidemic came later and slower as *d* increased. The cumulative number of infected students became larger and the time to reach saturation came later as *d* increased. None of the simulation curves in infected students replicated the two remarkable peaks.

**Fig 5:**
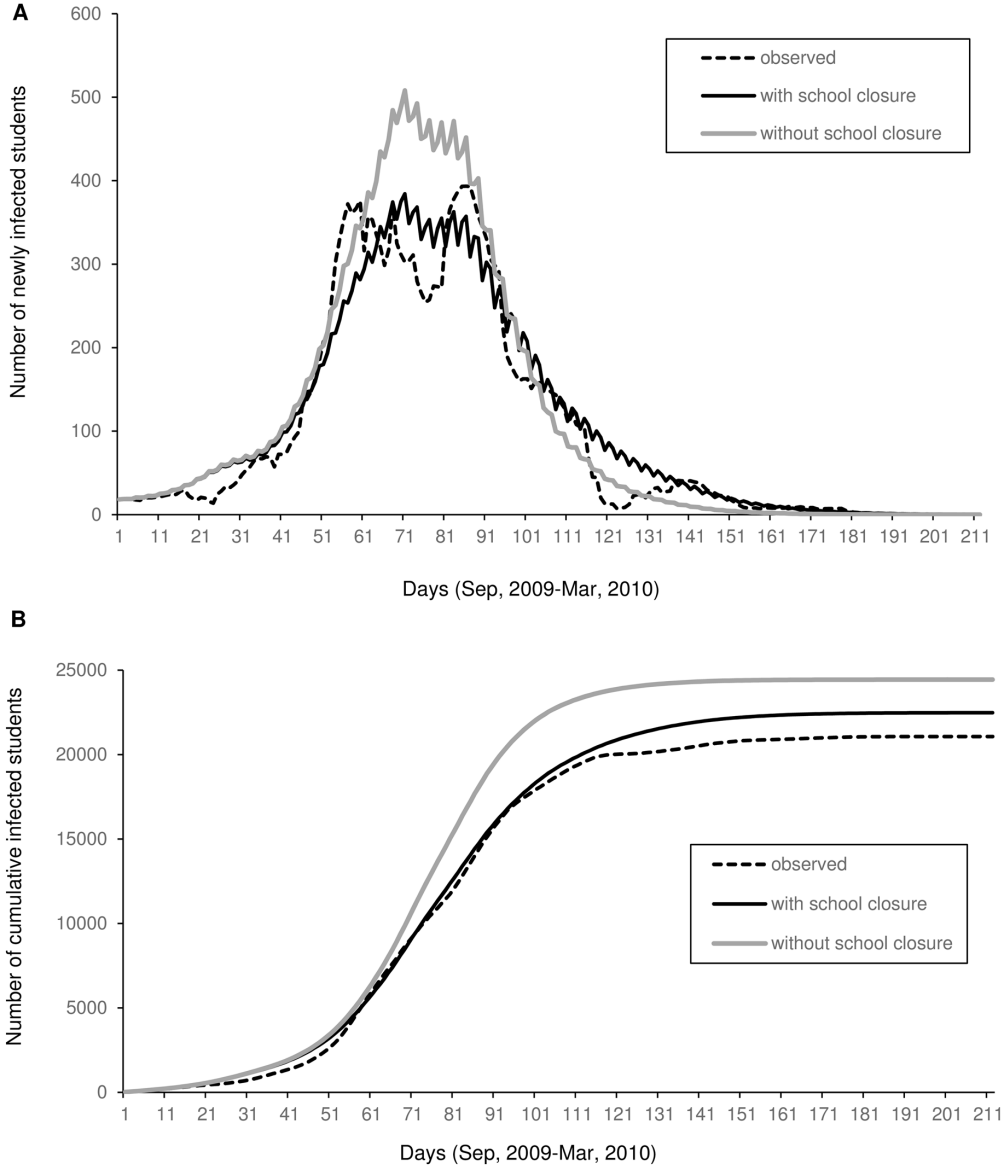
Simulated time course with and without school closure in *d* = 3 in Oita City, Japan. From September 2009 to March 2010, (A) the number of newly infected students and (B) cumulative number of infected students.

We calculated the correlation coefficient to determine *d* between the observed and simulated values of the number of infected students and the cumulative number of infected students (Table 2). Every correlation coefficient was high, but correlation coefficients of both infected students and the cumulative number of infected students in the case of *d* = 3 were the highest. Therefore, we adopted partial regression coefficients in the case of *d* = 3 in the simulation. We compared the simulations of infected students in a school closure using Eq (2) with the simulation without a school closure using Eq (1) in *d* = 3 (Fig 5). The number of infected students in a school closure decreased by 24% at its peak, and the cumulative number of infected students decreased by only 8.0%. The peak in the curve both with school closure and without school closure occurred at the same time in early November 2009. The time when the number reached 99% of the total number of infected students was observed later with school closure.

**Table 2:** Relationship between observed and simulation values by Pearson’s correlation coefficient test.

	Newly infected students		Cumulative number of infected students	
Delay	*r*	*P* value	*r*	*P* value
*d* = 1	0.741	<0.001	0.983	<0.001
*d* = 2	0.922	<0.001	0.994	<0.001
*d* = 3	0.964	<0.001	0.999	<0.001
*d* = 4	0.913	<0.001	0.992	<0.001
*d* = 5	0.817	<0.001	0.980	<0.001

## Discussion

In this study, we discuss the evaluation of our model, the meaning of the time delay, and the impact of school closure as demonstrated by simulation.

### Evaluation of our model

Our model can provide a useful basis for simulating the observed outbreak and evaluating possible preventive measures for mitigating the spread of influenza. We evaluated the effect of school closure by analysing daily reports of school closure and the number of cases in every public school in Oita City during pandemic (H1N1) 2009 using a regression model involving time delay, the influence of school closure, and AH. The delay was between infection and becoming infectious (Fig 1). We estimated this length of delay as three days using the *R* ^2^ of observed and simulated values (Table 2). This result was consistent with a common latent period of 2–3 days [39]. We showed our simulation of the number of students infected daily and the cumulative number of infected students in different time delays in Fig 4. At the onset until mid-October 2009, the best fitting curve seemed to be the curve of simulation in *d* = 3. It seems that the most reasonable time delay is 3 days. In contrast, the cumulative number of infected students in *d* = 3 was larger than that observed. Because our model was based only on the data before winter vacation, the influence of winter vacation was not properly reflected in the model. In addition, the reported number of cases during a long vacation might be underreported; therefore, the true extent of infection is unknown.

In the observed curve, the number of infected students had two notable peaks, whereas in our simulation, the two peaks were faint (Figs 2 and 4). The same pattern was also observed in the curves of children aged 4–6 years, 7–9 years, and 10–14 years (Fig 3). The number of infected students increased rapidly at the end of October 2009 and decreased quickly to mid-November 2009 (Fig 2B). The cause of this reduction may be due to many school closures implemented at the end of October 2009. The pattern in our simulation was attributable to the fact that our simulation curve in the case of *d* = 3 was not able to replicate the rapid increase in October.

Other studies have reported multiple peaks in one season. In Iki City, Nagasaki, Japan, an epidemiological study reported two outbreaks among schoolchildren in pandemic (H1N1) 2009 [24]. This study concluded that the two outbreaks were caused by school closure, and school closure was considered very effective in controlling the spread of the pandemic. Although the duration of suspension established by Iki City (7 days for an infected student and 5 days for students in close contact with the infected student) was different from that in Oita City, their findings are useful to assess the effect of school closure. In the 1918 pandemic in the United Kingdom, there were three waves of mortality patterns [41]. One study concluded that the factor of three waves was caused by schools opening and closing, temperature changes, and changes in human behaviour. However, the behavioural responses had the largest effect. Our model included the influence of weather as AH. It was reported that AH is related to the onset of the epidemic, and AH decreased below 11 g/m^3^ at the onset of influenza epidemics in Japan [40]. AH decreased below 11 g/m^3^ at the end of September 2009. This timing is the same as the beginning of the increase in the number of infected students (Fig 2C). AH was almost maintained at <11 g/m^3^ after the beginning of October 2009. In Table 1, the partial regression coefficient, *β*_1_, indicated that the degree of AH was lower and the number of infected students was larger. These findings suggested that AH is related to the beginning of the increase in the number of infected students.

### Meaning of the time delay

We designed the regression model from the SEIR model by incorporating a time delay (Fig 1). The best fitting case was *d* = 3 (Table 2). Transmissions reported as a case at *t*+1 occurred as infected at *t*−3 (Fig 1). Therefore, because we used fixed intervals for the latent and infectious periods in our model to fit the moving average, the influence of infection continued for an average of approximately 4 days. This finding suggests that school closures lasting more than 4 days effectively decreases the number of infected students at school. Our finding may help principals determine the duration of school closure and the timing for re-opening the school.

In Japan, the optimal duration of reactive class closure has been investigated in several studies. Sugiura et al. reported that the minimum effective number of days for a class to be closed was 5 days. They did not refer to the timing [23]. Yamamoto et al. recommended class closure for ≥6 days when the number of infected students reached >20% of the class [25]. The statistical approach compared different durations of school closure based on absentee data, which might introduce bias for shorter durations because the number of absent students decreases after the duration for recovery is reached. There are several approaches to determine the duration of school closure. Thus, we should establish methods of evaluation before reaching a unified conclusion.

### Effect of school closure illustrated by simulation

We simulated the daily number of infected students and counted the cumulative number of infected students with and without school closure. School closure decreased the number of infected students at the peak by as much as 24% and the number of cumulative infected students by only 8.0% (Fig 5). These results were based on the reactive school closure strategy under the established rule during the 2009/2010 season. Generally, there are three measures to mitigate damage of influenza: 1) delay the peak, 2) reduce the number of cases at the peak, and 3) reduce the number of cumulative patients. Our results were based on the second measure. In other studies of proactive school closure for a pandemic that used mathematical models, school closure was remarkably effective for decreasing the number of infected students at the peak, but it was less effective in decreasing the total number of infected students [8–10]. In contrast, the reduction in the number of infected students at the peak in our study was minimal. Several studies have shown that reactive school closure reduces the number of infected cases within the class [22, 23, 25, 42, 43]. Therefore, reactive school closure was probably more effective for prevention within the class but less effective for prevention within the whole region.

We have to note that the epidemic became prolonged in our simulation. If a delay in the peak and a reduction in the cumulative number of infected students must be achieved, a different type of school closure strategy should be adopted. The Hyogo and Osaka prefectures implemented significant school closures proactively during pandemic (H1N1) 2009. The prefecture-wide school closure strategy may have an effect from the viewpoint of a delay in the peak [16]. Therefore, we should decide the most suitable strategy of school closure that is in agreement with the goal of the closure.

Our study has several limitations. First, we only analysed the spread of influenza within schools disregarding interaction between students and people within the community. Students under school closure may have contact within families and community [37, 38]. Second, our analysis also did not examine the age of students and various behaviours based on different ages. A detailed analysis is desirable to provide a reliable prediction model, but involving such factors may complicate the analysis. Third, we disregarded classes and treated the population of school children as a whole. For more detailed analyses, a class level model of transmission dynamics is required. We believe the results presented here are useful and will contribute to the next step of conducting a more detailed analysis.

## Conclusions

We evaluated the impact of school closure by analysing daily reports of school closure and the number of cases in every public school in Oita City in pandemic (H1N1) 2009 using a regression model involving a time delay. School closure was an effective intervention for mitigating the spread of influenza. Our model included the delay between infection and becoming infectious. The best fitting delay was 3 days. This result suggests that school closure should be implemented for more than 4 days. School closure has a remarkable effect in decreasing the number of infected students at the peak, but it is less effective in decreasing the total number of infected students.
